# Systematic Review of Scalable Biomarkers of Alzheimer’s Disease

**DOI:** 10.1002/alz.087415

**Published:** 2025-01-09

**Authors:** Shivani Suresh, Vanessa Raymont, Sarah Bauermeister

**Affiliations:** ^1^ University of Oxford, Oxford United Kingdom; ^2^ Dementias Platform UK ‐ University of Oxford, Oxford United Kingdom

## Abstract

**Background:**

Alzheimer's disease (AD) impacts over 50 million individuals and imposes a substantial burden on patients, caregivers, and society at large. Recent research suggests that AD is a continuum comprising preclinical, prodromal, and dementia stages, with underlying pathology manifesting well before symptoms appear. Early and accurate diagnosis is therefore crucial for optimal clinical outcomes; yet current diagnostic methods, such as neuroimaging and cerebrospinal fluid lumbar puncture, are expensive and invasive. This highlights the need for more scalable and cost‐effective alternatives, particularly blood biomarkers. In addition to their diagnostic potential, these biomarkers can also screen at‐risk individuals and offer valuable prognostic insights. Therefore, a systematic review of existing literature on scalable AD blood biomarkers is essential to address the current literature gap and highlight advancements in the field.

**Method:**

To conduct this systematic review, a comprehensive search of the literature will be performed across multiple databases, including PubMed, EMBASE, Global Health, Journals@OVID, and PsycINFO. In light of the recent NIA‐AA recommendations, focus will be laid on articles assessing the clinical utility of plasma phosphorylated tau‐217 (p‐tau217), glial fibrillary acidic protein (GFAP), and neurofilament light chain (NfL). The search criteria will be limited to human studies and English‐language journal articles published within the last 5 years. The review will include a qualitative synthesis of selected studies, and if feasible, a random‐effects meta‐analysis may be conducted. The meta‐analysis will pool studies based on the specific biomarkers under assessment to provide a quantitative overview of findings.

**Result:**

The systematic review aims to highlight advancements in scalable AD biomarkers, with a specific focus on plasma p‐tau217, GFAP, and NfL. An initial search of the literature brought up 3086 papers. After removing duplicates and conducting a preliminary screening, 159 articles have been selected for review. Through the qualitative synthesis of selected studies and potential meta‐analysis, the review intends to provide a comprehensive overview of the clinical utility of these biomarkers.

**Conclusion:**

This review will contribute to laying the foundation for improved clinical care practices in AD by summarizing key findings, identifying gaps in the literature, and outlining future applications of blood biomarkers in the diagnosis and management of AD.

